# Eating Disorders Impact on Vigilance and Decision Making of a Community Sample of Treatment Naive Attention-Deficit/Hyperactivity Disorder Young Adults

**DOI:** 10.3389/fpsyt.2018.00531

**Published:** 2018-11-06

**Authors:** Bruno Palazzo Nazar, Amanda Pompeu Trindade, Monica Leslie, Leandro Fernandes Malloy-Diniz, Joseph Sergeant, Janet Treasure, Paulo Mattos

**Affiliations:** ^1^Institute of Psychiatry, Federal University of Rio de Janeiro, Rio de Janeiro, Brazil; ^2^Institute of Psychiatry, Psychology and Neuroscience, King's College London, London, United Kingdom; ^3^Departamento de Saúde Mental, Universidade Federal de Minas Gerais, Belo Horizonte, Brazil; ^4^VU University Amsterdam, Amsterdam, Netherlands; ^5^D'Or Institute for Education and Research, Rio de Janeiro, Brazil

**Keywords:** ADHD, eating disorders, comorbidity, neuropsychology, bulimia, binge eating, obesity, decision making

## Abstract

Although impulsivity is suggested as a possible link to explain the association of Attention-Deficit/Hyperactivity Disorder (ADHD) with an Eating Disorder (ED), there is little research on how clinical and cognitive/neuropsychological functioning might change when this comorbidity occurs. ADHD individuals are at a higher of developing ED and also obesity. Some research has described the impact of ADHD in clinical treatment-seeking samples of ED patients. Consequently, we investigated how ED impacted on clinical and cognitive variables of a community sample of treatment-naive ADHD individuals. Ninety college students arranged in three groups (ADHD+ED, ADHD only and Controls) were analyzed using semi-structured interviews for ADHD (K-SADS), the Iowa Gambling Task, the Conner's Continuous Performance Test, Digit and Visual span, as well as rating scales for anxiety (STAI), depression (BDI) and impulsivity (BIS-11), and binge eating (BES). We found that ADHD+ED individuals significantly differed from both groups, presenting with a higher body mass index; more hyperactivity-impulsivity symptoms; higher binge eating scores; more omission errors on the Continuous Performance Test; disadvantageous choices on the Iowa Gambling Task. Also, we demonstrated through a moderation/mediation analysis that a greater level of binge eating mediated the increases in body mass index on our sample. There were no significant paths to explain binge-eating severity through changes on any of the neuropsychological tests used. The presence of an ED in normal weight in a community sample of ADHD individuals is associated with higher body mass index and a worse cognitive functioning.

## Introduction

A recent meta-analysis found that the risk of diagnosing an Eating Disorder (ED) in patients with Attention-Deficit/Hyperactivity Disorder (ADHD) is 3.82 times greater when compared to the general population (1). The heightened risk remains significant after controlling for age and gender, and holds true for all eating disorders syndromes [Anorexia Nervosa (AN), Bulimia Nervosa (BN), and Binge eating Disorder (BED)]. Furthermore, even though EDs are 10 times more prevalent in females, the association is significant for both sexes (2). Interestingly, even before full ED syndromes have developed at adolescence, eating disorder symptoms (e.g., loss of control eating) have also been significantly associated with ADHD (3, 4).

The comorbidity of ADHD with ED is of interest as it defines a subgroup of patients who have greater disordered eating habits (5) and might respond differently to current standard treatments for EDs (6). These patients are also at risk of presenting with a more disrupted mental functioning, exemplified by higher rates of other psychiatric comorbidities, especially greater rates of substance abuse (5, 7). ADHD symptoms can also indicate a higher severity of eating disorder symptoms and personality psychopathology in ED patients (8). Although this has been demonstrated in clinical samples (5, 9), there is scarce evidence to generalize the same phenomenon for community samples. Eating disorders respond poorly to treatment after a 1 year follow up if ADHD symptoms are present at baseline, especially if they present with high inattention symptoms (10).

Of note, the recent approval of lisdexamphetamine for the treatment of Binge Eating Disorder in the United States (11) suggests that psychostimulant medication might have a direct effect on eating behavior regardless of ADHD status. However, this is still poorly understood and concern may arise that populations at-risk for developing an eating disorder might misuse psychostimulants seeking weight regulation or as a compensatory behavior to their disordered eating habits (12, 13).

Results from studies evaluating how comorbid mental disorders influence cognitive functioning of ADHD have had varying results for children (14, 15) and adults (16, 17). Also, most studies have focused on anxiety and mood disorders. The diverse cognitive domains investigated and the varying results for different comorbid disorders prevent us from defining a specific profile of how a comorbid disorder impairs cognitive functioning in ADHD.

Two previous studies explored neuropsychological differences of ADHD individuals comorbid with an eating disorder. Seitz et al. (18) compared a sample of adult women with BN and a past history of ADHD (*n* = 12) to those without a history of ADHD (*n* = 45). They found that women comorbid for BN with ADHD presented more pronounced inattention and impulsivity when compared to those with BN only. This study also found that inattention was significantly more associated with these deficits than hyperactivity/impulsivity. One other study investigated if neuropsychological measures could explain the association of ADHD symptoms with BED symptoms. Steadman et al. (19) assessed 44 individuals and reported that impulsivity measured through a Continuous Performance Test (CPT) didn't moderate the correlation of ADHD and Binge Eating symptoms. The scarce literature on the causal pathways to explain this comorbidity have pointed toward impulse regulation deficits but further studies are necessary to corroborate this hypothesis (20).

In the present work, we aimed to test whether the presence of an ED was associated with poorer attentional function and decision-making in individuals with ADHD. Also, as a secondary aim, we tried to replicate findings from studies that evaluated the clinical profile across these disorders, in a community and treatment-naive sample.

## Materials and methods

### Sample

The present study was an analysis of a larger protocol presented in detail elsewhere in an open access manuscript (21), hereon summarized. A sectional study was conducted using a convenience sample from the fifth year of the medical course from the Federal University of Rio de Janeiro (UFRJ), over four consecutive years (2010–2014 with 8 recruitment waves in total). All students were invited to participate protocol when they initiated the fifth year, during the first class of psychiatry. The protocol was approved by the Institute of Psychiatry—UFRJ Ethics Committee.

All participants provided informed consent to take part, and the Institute of Psychiatry of the Federal University of Rio de Janeiro (IPUB-UFRJ) Ethics Committee approved this study. The exclusion criteria for the present analysis were: Presence of any psychiatric diagnosis other than ADHD or ADHD+ED, epilepsy, and current use of any psychotropic medication.

### Procedures

#### Screening

Participants were screened for participating in the research with:

- Adult Self-Rating Scale (ASRS-18) (22): an 18-item self-report questionnaire used as a screening tool for ADHD.
- *Binge Eating Scale* (BES) (23): a 16-item self-report questionnaire used as a screening tool for binge eating, which also evaluates the severity of binge eating.

#### Clinical assessment

All participants were evaluated through semi-structured interviews using DSM-5 criteria. These evaluations were completed by board certified psychiatrists with more than 10 years of clinical experience in adult ADHD and ED (BPN and PM). All diagnosis were discussed using history, self-report, and semi-structured interviews.

The K-SADS interview adapted for adults with ADHD (24) was used to diagnose ADHD. The participant received a diagnosis of ADHD if they met DSM-5 criteria for at least 5 *current* inattention *or* hyperactivity/impulsivity symptoms, associated with at least 5 *past* inattention *or* hyperactivity/impulsivity symptoms, with onset before the age of 12 and occurring in at least 2 life-time domains with significant impairment. All ADHD assessments were done blind to the participant's self-report status, and all students with more than 5 current symptoms of inattention or hyperactivity/impulsivity were discussed with the other rater for consideration of a diagnosis.

- SCID-P module for ED.
- MINI-Plus: for all other psychiatric diagnosis.

Participants completed the following questionnaires:

- *Beck Depression Inventory* (BDI) (25, 26) developed for assessing the severity of depressive symptoms. The BDI contains 21 questions, which are rated on a Likert scale ranging from 0 to 3.
- *State-Trait Anxiety Inventory* (STAI) (27) is composed of two 20-item scales that measure trait and situational anxiety.
- *Barrat-Impusivity Scale* (BIS-11) (28): This scale measures impulsivity in life situations. The original BIS-11 uses three subscales but the Brazillian transcultural adaptation studies validated two subescales—which were used for the present analysis—in two domains: attentional/planning (BIS-ATPLAN), cognitive and motor inhibition (BIS-CINI) impulsivity.
- All the mentioned rating scales have been translated and adapted to Brazilian Portuguese [ASRS-18 (29), BES (30), BDI (26), STAI (27) and BIS-11(31)].

#### Neuropsychological assessment

Neuropsychological evaluation comprised:

- **IQ**, calculated with the four-subtest form of the Wechsler Abbreviated Intelligence Scale (WASI) (32), from which the blocks, vocabulary, matrix, and similarities tests were administered.
- The **Digit Span** and the **Visual Span**: Both these tests are used to assess executive functioning. In the verbal Digit Span task the subject has to recall forward and reverse orders of digit sequences. It uses the phonological loop to measure working memory, attention, and inhibition. The Visual Span is a visuospatial version of the verbal span in which the subject has to recall forwards and reverse sequences of a visual task (tapping on cubes).
- The **Conner's Continuous Performance Task II (CPT-II)** (33): Continuous performance tests are the most commonly used attention tasks in clinical practice (34) and give the opportunity to evaluate the ability of a subject to maintain consistent responses over time and speed of stimuli presentation (35). Variables of interest in the present study were: number of omission errors (OMI), commission errors (COM), hit reaction time (HRT), HRT block change (HRT BL CHANGE), reaction time by inter-stimulus interval (Hit RT ISI CHANGE), and attentiveness (d′). OMI errors occur when subjects fail to respond on trials containing target letters (all non-“X” letters), COM errors occur when they respond on trials with letters “X.” HRT is the mean response time for all non-X responses over all six time blocks and represents the subject's easier discrimination of the target. HRT BL CHANGE (a vigilance measure) is the slope of change in the reaction time over the six time blocks; a positive slope indicates a slowing RT, and a negative slope indicates a quicker RT as the test progresses. Hit RT ISI CHANGE (capacity to adjust to presentation speed) is calculated by computing the slope of change in RT over the three ISIs (1, 2, and 4 s). The ISIs are block-randomized so that all three ISI conditions occur every block but in a different order; by varying the inter-stimulus intervals (1, 2, and 4 s), it is possible to assess the ability to adjust to changing *tempo* and task demand. The index d′ reflects the subject's perceptual sensitivity to targets as a measure of how well the individual discriminates between targets (signals) and non-targets (noise). Higher d′-values indicate greater sensitivity and better discrimination between targets and non-targets.
- The **Iowa Gambling Task (IGT)** (36): We used a computerized version of the IGT, in which subjects had to choose a card from four decks. They were told that in order to win the largest sum of money some decks were advantageous while others were disadvantageous. Two decks brought large immediate gains with large future losses (decks A and B), while two decks lead to small wins but also small future losses (Decks C and D). After 100 trials, a net score was calculated using the equation [(Decks C+D) – (Decks A+B)], which produces a measure of the total number of advantageous decks minus the total number of disadvantageous decks. This was also calculated for 5 blocks of 20 trials, which enables the assessment of learning over the test. This test measures decision making and non-planning impulsivity.

### Sample

A total of 726 students were eligible for the study but only 662 (91.1%) were screened using the ASRS-18 and BES as some students were not present during research presentation and screening procedures. From the 662 screened students, board certified psychiatrists using the semi-structured interviews interviewed a total of 344 students for mental disorders. All students with positive ASRS-18 or BES were interviewed as well as a random equal proportion of negative screenings. The final sample analyzed in the present study final sample consisted of 90 students. There were no statistically significant differences between the age and gender profile of protocol completers and non-completers.

### Statistical analysis

Statistical tests were performed using SPSS v.20. Subjects were classified into one of three diagnostic groups (Control, ADHD only, ADHD+ED). We have included subjects in the “Control” group that didn't have any psychiatric diagnosis assessed using the MINI-Plus and didn't fulfill criteria for either ADHD or ED. In the ADHD group we included subjects that fulfilled criteria for ADHD but didn't have ED. For the ADHD+ED group we included patients presenting the comorbidity with both diagnoses. The differences between groups were considered significant if *p* < 0.05. The demographic characteristics were tested across groups using a one-way analysis of variance (ANOVA). Afterwards, pairwise contrasts were obtained using independent-samples *t*-tests. *Post-hoc* with Fisher's LSD correction was used.

For the mediation analysis we used the *paramed* command from STATA (37). The significance of the indirect and total effects was tested via bias-corrected bootstrapping, which is robust to violations of the assumption of homoscedasticity.

## Results

### Sample characteristics

The sociodemographic characteristics for the final sample are detailed in Table 1. The three diagnostic groups were proportional and had non-significant differences regarding gender distribution (*p* = 0.215), socioeconomic status (*p* = 0.976), and global IQ (*p* = 0.46). None of the ADHD subjects from the ADHD-only or from the ADHD+ED groups were currently taking any medication.

**Table 1:** Subjects sociodemographic and clinical characteristics.

	**Total Sample (*n* = 90)**	**Controls (*n* = 39)**	**ADHD (*n* = 35)**	**ADHD+E (*n* = 16)**	***p*-value^a^**	**Effect-Size (Partial Eta^2^)^a^**
Age	23.71 (±1.9)	23.3 (1 ± 0.2)	24 (±2.3)	24 (±1.6)	0.215	0.035
Gender: % (*n*)					0.976	0.001
Female	81% (68)	81.8% (27)	80% (28)	81.3% (13)		
Weight, kgs	62.6 (±12.2)	60.2 (±9)	60.5 (±9.7)	73.2 (±17.5)	<0.0001^b^	0.167
B.M.I.	22.37 (±3.4)	21.6 (±2.7)	21.8 (±2.8)	25.7 (±4.3)	<0.0001^c^	0.205
Overweight	13% (*n* = 11)	10.4% (4)	8.7% (3)	37.8% (6)		
Obese	2.3% (*n* = 2)	0% (0)	2.9% (1)	6.3% (1)		
SES: % (*n*)					0.184	0.038
≥ 32,500 $/y (A1)	6.7% (*n* = 6)	7.7% (3)	8.6% (3)	0% (0)		
≥ 21,900 $/y (A2)	11.1% (10)	2.6% (1)	20% (7)	12.5% (2)		
≥ 6,800 $/y (B 1 e 2)	30% (27)	28.2% (11)	28.6% (10)	37.5% (6)		
≥ 2,500 $/y (C e D)	52.2% (*n* = 47)	61.5% (24)	42.9% (15)	50% (8)		
Global IQ	113 (9)	112 (10)	113 (8)	116 (8.2)	0.46	0.021

a Univariate Analysis. Omnibus p-values and effect sizes. LSD post-hoc correction.

b Control = ADHD, p = 0.889; Control < ADHD + ED and ADHD < ADHD + ED, p < 0.001.

c *Control = ADHD, p = 0.786; Control < ADHD + ED and ADHD < ADHD + ED, p < 0.001*.

The ADHD+ED group consisted of five participants with bulimia Nervosa; three participants with BEDs; three participants with subclinical Bulimia Nervosa (didn't fulfill the frequency criteria for binge and purging episodes); and five participants with subclinical BED (didn't fulfill the frequency criteria for binge episodes).

The proportion of obese and overweight participants in the ADHD+ED group was significantly higher when compared to both the ADHD only and to the Control groups (*p* = 0.004). Also, the mean BMI in this group was significantly greater than the other two groups: 4.1 points higher than the control group and 3.9 points higher than ADHD-only group. The ADHD+ED group was 13 kg (28.6 lbs) heavier than the control group and 12.7 kg (28 lbs) heavier than ADHD-only group on average (Table 1).

The analysis of ADHD symptoms revealed that the ADHD+ED group had significantly greater current Hyperactivity/Impulsivity than the ADHD only group. All other comparisons were non-significant between the ADHD+ED and the ADHD only group, with a trend for greater past Inattention symptoms in the ADHD+ED group (Table 2).

**Table 2:** ADHD symptoms and self-report psychopathological measures.

	**Total Sample (*n* = 90)**	**Controls (*n* = 39)**	**ADHD (*n* = 35)**	**ADHD+ED (*n* = 16)**	***Omnibus p*-value^a^**	**Contrasts**	***Omnibus* Effect-Size (Partial Eta^2^)^a^**
Current Inatt	N.A.	1.5 (±1.9)	5.9 (±1.5)	6.8 (±1.3)	<0.001	1<2=3^b^	0.657
Current H/I	N.A.	1.2 (±1.2)	4 (±2.2)	5.6 (±2.7)	<0.001	1<2<3^c^	0.433
Past Inatt	N.A.	1.1 (±1.3)	5 (±1.9)	6.1 (±1.9)	<0.001	1<2=3^d^	0.607
Past H/I	N.A.	1.1 (±1.4)	4.2 (±2.4)	4.4 (±2.8)	<0.001	1<2=3^e^	0.349
BIS - Total	66.6 (±12)	58.9 (±8.8)	71.8 (±10.3)	71.4 (±15.8)	<0.001	1<2=3^f^	0.256
BIS-ATTPL	18.3 (±4.1)	17.9 (±4.7)	18.8 (±3.9)	18 (±3.6)	0.73	1 = 2 = 3	0.010
BIS-CINI	43.5 (±8.4)	36 (±4.8)	48 (±6.6)	50 (±5.2)	<0.001	1<2=3^g^	0.547
STAI-T	41.7 (±9.9)	38.4 (±9.4)	44.2 (±9.6)	44.6 (±9.8)	0.032	1<2^h^	0.089
STAI-S	42.2 (±10.4)	38 (±8.7)	45.3 (±11.5)	44.5 (±8.1)	0.23	1<2^i^	0.087
BES	8.7 (6.3)	6,22 (5.3)	8.82 (6)	15 (5.3)	<0.001	1=2<3^j^	0.234
BDI	7 (±6.9)	4.5 (±4.5)	8.8 (±7.2)	8.5 (±9.1)	0.03	1<2=3^l^	0.092

Mean (SD). N.A, Not Applicable; Current Inatt, KSADS Current Inattention symptoms; Current H/I, KSADS.Current Hyperactivity/Impulsivity symptoms; Past Inatt, KSADS Past Inattention symptoms; Past H/I KSADS Past Hyperactivity/Impulsivity symptoms; BIS, Barrat Impulsivity Scale; BIS-ATTPLAN, Attention and Planning BIS subscale; BIS-CINI, Inhibitory Control BIS subscale; STAI-T and –S, State-Trait Anxiety Inventory – Trait and –State; BES, Binge Eating Scale; BDI, Beck depression inventory.

1 = Controls; 2 = ADHD; 3 = ADHD+ED.

a Univariate Analysis. Omnibus p-values. LSD post hoc correction.

b 1 < 2, p < 0.001; 1 < 3, p < 0.001; 2 = 3, p = 0.11.

c 1 < 2, p < 0.001; 1 < 3, p < 0.001; 2 < 3, p = 0.007.

d 1 < 2, p < 0.001; 1 < 3, p < 0.001; 2 = 3, p = 0.53.

e 1 < 2, p < 0.001; 1 < 3, p < 0.001; 2 = 3, p = 0.75.

f 1 < 2, p < 0.001; 1 < 3, p = 0.002; 2 = 3, p = 0.92.

g 1 < 2, p < 0.001; 1 < 3, p < 0.001; 2 = 3, p = 0.34.

h 1 < 2, p = 0.01; 1 = 3, p = 0.052; 2 = 3, p = 0.89.

i 1 = 2, p = 0.059; 1 = 3, p = 0.065; 2 = 3, p = 0.69.

j 1 = 2, p = 0.07; 1 < 3, p < 0.001; 2 < 3, p = 0.002.

l *1 < 2, p = 0.025; 1 < 3, p = 0.031; 2 = 3, p = 0.757*.

### Self-report psychopathology scales

Regarding anxiety symptoms, there were no significant differences on state anxiety (*p* = 0.23) and there was a significant difference on trait anxiety between controls and ADHD only (*p* = 0.01) or between controls and ADHD+ED (*p* = 0.05). The ADHD only and ADHD+ED groups didn't present significant differences on state (*p* = 0.69) or trait (*p* = 0.89) anxiety. The same occurred with depressive symptoms. Although controls had significantly lower BDI score than ADHD only (*p* = 0.02) and than ADHD+ED (*p* = 0.03), the last two groups didn't differed significantly among themselves (*p* = 0.75). In the analysis of self-report impulsivity, only the BIS-Total (*p* < 0.001) and BIS-CINI (*p* < 0.001) scores presented significant differences with ADHD only and ADHD+ED groups having higher scores than the control group.

### Neuropsychological assessment

**Figure 1:**
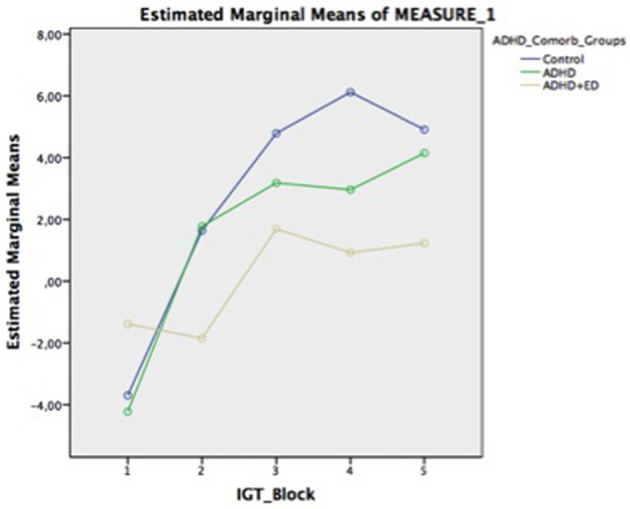
Iowa Gambling Task across groups over time-in-task.

Results are reported by means and standard deviations for each group and total, for each test, in the Supplementary Materials. Hereon are reported mean differences and group comparisons with *post-hoc* tests. The neuropsychological assessment of verbal and non-verbal IQ (Table 1), Digit and Visual Span (Table 3), and the IGT (Table 4) did not show significant differences across groups. However, there was a trend for the ADHD+ED group having more disadvantageous choices than the other two groups across block 2 and in the net score of the IGT (Figure 1). Of note, only the deck B measure from the IGT was found to be to be significantly different in the ADHD+ED vs. Controls (*p* = 0.05).

**Table 3:** Interactions among groups for digit and visual span.

		***Omnibus F (Eta^2^), p-value^a^***	**ADHD vs. Control**	**ADHD+ED vs. Control**	**ADHD+ED vs. ADHD**
			**Mean Difference (Cohen's d)**	**Mean Difference (Cohen's d)**	**Mean Difference (Cohen's d)**
Digit span	Raw score	0.93 (0.02), *p* = 0.39	−1.38 (1.87)	0.19 (−0.20)	1.19 (−1.24)
	Straight sequence	0.83 (0.02), *p* = 0.44	−0.72 (1.75)	−0.05 (0.09)	0.67 (1.24)
	Higher straight sequence	0.57 (0.01), *p* = 0.56	−0.19 (.82)	0.27 (−0.93)	0.46 (−1.53)
	Reverse sequence	0.63 (0.01), *p* = 0.53	−0.65 (1.56)	−0.13 (0.26)	0.51 (−0.93)
	Higher reverse sequence	0.73 (0.02), *p* = 0.48	−0.31 (1.33)	0.19 (−0.58)	0.50 (−1.58)
Visual span	Raw score	0.13 (0.004), *p* = 0.87	−0.36 (0.74)	−1.42 (0.22)	0.22 (−0.34)
	Straight sequence	0.004 (0.000), *p* = 0.99	0.93 (0.13)	0.00 (0)	0.03 (0.10)
	Higher straight sequence	0.17 (0.005), *p* = 0.83	0.09 (−0.46)	−0.12(.47)	−0.21 (0.82)
	Reverse sequence	0.30 (0.009), *p* = 0.73	−0.32 (1.13)	−0.14 (.37)	0.18 (0.49)
	Higher reverse sequence	0.56 (0.01), *p* = 0.57	−0.26 (1.52)	−0.09 (.41)	0.16 (−0.76)

Mean difference (Cohen's d).

a *Univariate Analysis. Omnibus p-values. LSD post-hoc correction*.

**Table 4:** Interactions among groups for Iowa gambling task.

	***Omnibus F (Eta^2^), p-value^*a*^***	**ADHD vs. Control**	**ADHD+ED vs. Control**	**ADHD+ED vs. ADHD**
		**Mean Difference (Cohen's d)**	**Mean Difference (Cohen's d)**	**Mean Difference (Cohen's d)**
IGT deck A	0.32 (0.009), *p* = 0.727	0.049 (−0.75)	1.63 (−0.29)	1.13 (−0.20)
IGT deck B	2.50 (0.067), *p* = 0.089	2.8 (−0.37)	5 (−0.71) ^*^	2.25 (−0.33)
IGT deck C	1.65 (0.045), *p* = 0.19	−2.46 (0.34)	−3.8 (0.34)	−1.33 (0.19)
IGT deck D	0.48 (0.014), *p* = 0.61	−0.61 (0.07)	−2.67 (0.35)	−2.05 (0.26)
IGT Block 1	0.90 (0.025), *p* = 0.41	−0.53 (0.08)	2.31 (−0.41)	2.83 (−0.44)
IGT Block 2	2 (0.054), *p* = 0.14	0.14 (−0.02)	−3.48 (0.69)	−3.62 (0.66)^a^
IGT Block 3	0.98 (0.027), *p* = 0.37	−1.6 (0.22)	−3.09 (0.41)	−1.49 (0.23)
IGT Block 4	2.12 (0.057), *p* = 0.12	−3.15 (0.35)	−5.19 (0.70)	−2.03 (0.29)
IGT Block 5	1.07 (0.03), *p* = 0.34	−0.76 (0.09)	−3.67 (0.53)	−2.91 (0.42)
Net score	2.12 (0.057), *p* = 0.12	−6.98 (0.30)	−13.77 (0.73)	−6.79 (0.37)^b^

Mean difference (Cohen's d).

* p < 0.05;

a p = 0.068;

b p = 0.053.

Univariate Analysis. Omnibus p-values. LSD post-hoc correction.

Decks A and B = disadvantageous; Decks C and D = advantegeous; Net score for all 100 trials = [(Decks C+D) – (Decks A+B)]; Block refers to Net score of only 20 trials.

In terms of vigilance testing, measures from the CPT (Table 5) yielded significant differences only for the Omission errors. There were significant differences when analyzing ADHD+ED vs. Controls (*p* = 0.031) and the ADHD+ED vs. ADHD only (*p* = 0.042). This difference was of a moderate effect size in both contrasts. All other CPT measures were non-significantly different in all comparisons (Table 5).

**Table 5:** Interactions among groups for Conner's continuous performance test.

	***Omnibus***	**ADHD vs. Control**	**ADHD+ED vs. Control**	**ADHD+ED vs. ADHD**
	***F (Eta^2^), p-value^a^***	**Mean Difference (Cohen's d)**	**Mean Difference (Cohen's d)**	**Mean Difference (Cohen's d)**
Omission	2.65 (0.072), *p* = 0.07	−0.02 (0.009)	9.05 (−0.42)^b^	9.07 (−0.42)^c^
Comission	1.45 (0.040), *p* = 0.24	2.56 (−0.31)	4.26 (−0.50)	1.69 (−0.19)
Standard error	0.85 (0.024), *p* = 0.43	0.24 (−0.002)	70.36 (−0.33)	70.11 (−0.32)
D Prime	1.74 (0.048), *p* = 0.18	−12.72 (0.29)	−25.41 (0.60)	−12.68 (0.29)
Variability	1.61 (0.045), *p* = 0.20	−85 (−0.89)	−304 (−2.54)	−218 (−1.76)
HRT	0.01 (0), *p* = 0.98	−285.13 (0.04)	−130.20 (0.02)	154.92 (−0.02)
HRT Block change	0.56 (0.016), *p* = 0.74	0.26(−0.29)	0.27(−0.21)	0.01 (−0.01)
HRT ISI change	0.29 (0.009), *p* = 0.74	0.26 (−0.09)	0.69 (−0.24)	0.42 (−0.09)

Mean difference (Cohen's d). HRT, Hit Reaction time; HRT ISI Change, HRT Inter Stimulus Interval Change.

a Univariate Analysis. Omnibus p-values. LSD post-hoc correction.

b p = 0.035.

c p = 0.041.

### Correlations between BMI, BES, BDI, and ADHD symptoms

Pearson correlations between BMI, BES, BDI, and ADHD symptoms (current KSADS inattention + current KSADS hyperactivity/ impulsivity as a single composite score) revealed that binge eating was positively correlated with depression (*r* = 0.025; *p* < 0.05) and ADHD (*r* = 0.43; *p* < 0.001) with a moderate effect size. Binge eating was positively correlated with BMI with a strong effect size (*r* = 0.48; *p* < 0.001). ADHD was positively correlated with depression (*r* = 0.33; *p* < 0.01) with a moderate effect size and didn't correlate with BMI (*r* = 0.23, *p* = 0.062). None of the correlations were so high as to suggestion redundancy (all correlations < |0.8|).

### Mediational analysis

The data were first analyzed for missing cases, outliers, and assumptions of normality. Seventeen cases were missing from the BES variable and eight cases were missing from the BDI variable. Cases with missing data on one or more variable were excluded from relevant analyses. One univariate outlier (*Z* > |3.00|) was observed in the BMI variable and one outlier was observed in the BDI variable. These cases were also excluded listwise from all further analyses. No univariate outliers were observed in the ADHD or BES variables. All variables were normally distributed (skew < |2.00|, kurtosis < |9.00|). The descriptive statistics associated with BES, BMI, BDI, and ADHD symptoms are reported in Table 6.

**Table 6:** Descriptive Statistics associated with binge eating, body mass index, depression, and ADHD symptoms.

	***M***	***SD***	**Min**	**Max**	**Skew**	**Kurtosis**
BES	8.86	6.42	0.00	22.00	0.38	−0.89
BMI	22.23	2.57	16.49	29.59	0.77	0.66
BDI	7.06	6.82	0.00	27.00	1.29	0.85
ADHD	7.19	4.86	0.00	17.00	0.18	−0.93

*BES, binge eating specific score; BMI, body mass index; BDI, Beck Depression Inventory; ADHD, current composite score of inattentive and hyperactive/impulsive symptoms. N = 69*.

Pearson correlations between BES, BMI, BDI, and ADHD symptoms are presented in Table 7. Binge eating was positively correlated with depression and ADHD (moderate effect size). Binge eating was positively correlated with BMI with a strong effect size. ADHD was positively correlated with depression (moderate effect size). None of the correlations were so high as to suggestion redundancy (all correlations < |0.8|).

**Table 7:** Correlations between binge eating, body mass index, depression, and ADHD symptoms.

	**BDI**	**BES**	**ADHD**
BES			0.43^***^
BDI		0.25^*^	0.33^**^
BMI	0.06	0.48^***^	0.23

BES, binge eating specific score; BMI, body mass index; BDI, Beck Depression Inventory; ADHD, current composite score of inattentive and hyperactive/impulsive symptoms.

N = 69.

* p < 0.05;

** p < 0.01;

*** p < 0.001.

Mediation model 1: Binge eating mediates the relationship between ED diagnostic status and BMI, after controlling for depression.

In the first mediation model we tested, we tested the hypothesis that binge eating mediates the effects of ADHD/ED comorbidity on BMI. This was investigated with a mediation analysis was conducted using the paramed command (37) in STATA (StataCorp, 2015). Assumptions of the mediation analysis for H1 were tested using two regression analyses in SPSS (IBM Corp., 2013): one bivariate regression of BES on eating disorder status and BDI (path a) and a multiple regression of BMI on BES, BDI, and eating disorder status (paths b′ and c′, respectively). Visual inspection of histograms of the standardized residuals revealed that they were approximately normally distributed for both regressions. Visual inspection of a scatterplot of standardized residuals plotted against standardized predicted values revealed that the assumption of homoscedasticity was violated for both regressions. A dummy regression did not reveal any multivariate outliers (Mahalanobis distance > 13.82, *df* = 2, *p* < 0.001) among the ED and BDI variables. A second dummy regression did not reveal any multivariate outliers (Mahalanobis distance > 16.27, *df* = 3, *p* < 0.001) among the ED, BDI, and BES variables. Neither regression was associated with excessive multicollinearity (tolerance > 0.10 for all predictor variables and covariates).

The mediation analysis was then conducted in STATA using the paramed command. The significance of the indirect and total effects was tested via bias-corrected bootstrapping, which is robust to violations of the assumption of homoscedasticity.

Both path a (coefficient = 7.86, SE = 1.76, 95% CI [4.34, 11.38] and path b′ (coefficient = 0.13, 0.05, 95% CI [0.04, 0.23]) were associated with significant but small positive effects. The analysis revealed that having an eating disorder was significantly positively associated with higher BMI (total effect = 3.31, bootstrap standard error = 0.63, 95% CI [2.06, 4.52]). Binge eating significantly mediated this effect (indirect effect = 1.04, bootstrap standard error = 0.58, 95% CI [0.09, 2.30]).

Mediation model 2: Impulsivity mediates the relationship between ADHD and binge eating, after controlling for depression.

For this mediation, we first sought to establish the existence of significant relationships between the predictor, mediator, and dependent variables. We therefore carried out a regression in which BES was entered as the dependent variable and the BDI, BIS, current KSADS inattention, and current KSADS hyperactivity/impulsivity were entered using the forced entry method. This model predicting binge eating from ADHD symptoms and impulsivity was significant, *R*^2^ = 0.21, Adj. *R*^2^ = 0.16, *SE* = 5.88, Δ*F*_(4, 60)_ = 9.64, *p* = 0.006. However, none of the included variables significantly predicted binge eating. The regression was not affected by multicollinearity (tolerance > 0.1 for all variables).

Therefore, given that a relationship between the mediator and dependent variable could not be established after controlling for the independent variable, the current model did not meet Baron and Kenny's necessary assumptions for a mediated model (38). While modern research has indicated that not all of Baron and Kenny's assumptions are necessary to establish a significant indirect effect (39), we concluded that an indirect effect in the absence of a significant effect between the mediator and dependent variable was not of theoretical interest in the current model.

## Discussion

In the present research we have demonstrated that individuals comorbid for ADHD and ED presented greater omission errors in the CPT and a tendency for impaired decision-making using the IGT. Also, these patients presented higher number of current Hyperactivity/Impulsivity symptoms than the ADHD-only group. Furthermore, we demonstrated that comorbid individuals had a higher BMI and that a greater level of binge eating mediated this relationship.

Previous research in the field of ADHD and Obesity has suggested that weight gain in individuals with ADHD might be due to a sleep disorder (e.g., sleep apnea) in obese patients mimicking ADHD (40), a genetic variant with impaired reward processing or altered eating habits due to impulsivity (41). We have presented evidence that the presence of a comorbid ED might contribute to weight gain in ADHD subjects. Even in the presence of mild eating binges, as measured by the BES, our ADHD+ED subjects had a significantly higher BMI than ADHD only. This falls in line with findings from clinical samples, where obese subjects presenting for weight loss comorbid with ADHD had higher BMI (5, 42–44).

The higher number of current HI symptoms in the ADHD+ED group cannot be regarded as an indication of higher impulsive traits or higher anxiety levels since these measures didn't differ from the other groups in self-report questionnaires. This contradicts the findings that ADHD-ED comorbid individuals had higher anxiety levels found in clinical samples of obese (5). ADHD individuals usually change their clinical presentation over time (45) with waning of HI behaviors to more socially age appropriate presentations.

Two previous studies have investigated cognitive function in participants with ADHD+ED. Reinblatt et al. (46), has investigated if children comorbid for Loss of Control Eating (a subclinical form of BED) differed from ADHD-only or Control participants using a Continuous Performance Test (The GNG neurobehavioural task) and a motor inhibition task. They didn't find any differences between groups. Furthermore, Seitz et al. (18) compared a sample of women with current bulimia nervosa with a history of childhood ADHD to a separate sample without a childhood history of ADHD on a continuous performance test, a task for divided attention, and a task for executive functioning. Although they didn't find significant results on the neuropsychological tests, there was a trend greater for omission errors in the comorbid group.

In accordance with the results found by Seitz et al. (18), our ADHD+ED group presented significantly higher omission errors on the CPT. Although this would indicate inattention, distractibility the number of current inattention symptoms didn't differ from ADHD only. Also, this index can denote a slower motor response. Apparently, the presence of an ED further impairs attentional systems in ADHD individuals.

The findings in the IGT suggest that ADHD+ED subjects have their decision-making skills impaired, when compared to ADHD only subjects. This might be explained by the presence of an ED. The IGT could be representative of an information processing mediated by the insula (47). A triadic model of impulse control postulates that abnormal functioning in different parts of the brain impairs this function. These cognitive systems control habitual and salient behaviors processed by the amygdala-striatum; self-regulation modulated by the prefrontal cortex; and translation of interoceptive states to feelings (urges, cravings) by the insula. It might be that ED impairs decision-making of ADHD individuals by the well-documented deficits of ED individuals in insular function (48, 49).

Binge eating was not significantly predicted by depression, impulsivity as measured by the BIS, inattention, or hyperactivity/impulsivity measured by the KSADS. Perhaps low sample size prevented the model from uncovering significant results. This effect might also reflect the poor ecological validity of these self-report measures, given the well-cited associated between impulsivity and binge eating (5, 18).

## Limitations

Our findings are limited by the small sample size, relatively mild-moderate severity of eating behavior disturbances. The high cognitive functioning of all samples, as they are college students, could interfere with cognitive testing as it could induce a ceiling effect of results. Differences could be more pronounced in a clinical sample with severe symptoms and a poorer cognitive functioning. On the other hand, our study has several strengths, exemplified by the control group without any mental disorder, and the use of a treatment-naive sample, as medication could be a factor that could interfere with cognitive testing.

## Author contributions

All authors listed have made a substantial, direct and intellectual contribution to the work, and approved it for publication.

### Conflict of interest statement

The authors declare that the research was conducted in the absence of any commercial or financial relationships that could be construed as a potential conflict of interest.
