# Supplementary material for: Eating Disorders Impact on Vigilance and Decision Making of a Community Sample of Treatment Naive Attention-Deficit/Hyperactivity Disorder Young Adults
Source: Front Psychiatry. 2018 Nov 6;9:531. doi: 10.3389/fpsyt.2018.00531 (PMC6232382; doi:10.3389/fpsyt.2018.00531)
Supplement: Supplementary file 1 [file Table_1.DOCX]

**Supplementary material.** Means and Standard deviation for Digit and Visual span.

|  |  | ***Total Sample*** | **Control Group** | **ADHD Group** | **ADHD+ED Group** |
| --- | --- | --- | --- | --- | --- |
|  | **Raw score** | 17.81 (4.08) | 18.42 (4.30) | 16.93 (3.60) | 18.23 (4.47) |
|  | **Straight sequence** | 10.21 (2.29) | 10.51 (2.25) | 9.75 (2.21) | 10.46 (2.60) |
| **Digit Span** | **Higher straight sequence** | 6.69 (1.29) | 6.72 (1.25) | 6.51 (1.21) | 7 (1.58) |
|  | **Reverse sequence** | 7.60 (2.31) | 7.90 (2.57) | 7.17 (1.94) | 7.76 (2.38) |
|  | **Higher reverse sequence** | 5.32 (1.35) | 5.42 (1.39) | 5.06 (1.19) | 5.61 (1.60) |
|  | **Raw score** | 17.59 (2.66) | 17.75 (2.86) | 17.39 (2.73) | 17.61 (2.06) |
|  | **Straight sequence** | 8.98 (1.65) | 9 (1.85) | 8.96 (1.42) | 9 (1.68) |
| **Visual Span** | **Higher straight sequence** | 6.13 (1.07) | 6.12 (1.11) | 6.21 (1.16) | 6 (.81) |
|  | **Reverse sequence** | 8.60 (1.62) | 8.75 (1.54) | 8.42 (1.93) | 8.61 (1.04) |
|  | **Higher reverse sequence** | 5.82 (0.95) | 5.93 (.96) | 5.67 (1.05) | 5.84 (.68) |
|  |  |  |  |  |  |
