# Supplementary material for: Eating Disorders Impact on Vigilance and Decision Making of a Community Sample of Treatment Naive Attention-Deficit/Hyperactivity Disorder Young Adults
Source: Front Psychiatry. 2018 Nov 6;9:531. doi: 10.3389/fpsyt.2018.00531 (PMC6232382; doi:10.3389/fpsyt.2018.00531)
Supplement: Supplementary file 2 [file Table_2.DOCX]

**Supplement.** Means (standard deviations) for Iowa Gambling Task.

|  | ***Total*** | **Control** **Group** | **ADHD Group** | **ADHD+ED Group** |
| --- | --- | --- | --- | --- |
| **IGT deck A** | 17.53 (6.16) | 17.06 (6.39) | 17.55 (6.69) | 18.69 (4.49) |
| **IGT deck B** | 27.87 (7.49) | 25.93 (7.84) | 28.74 (7.19) | 31 (6.19) |
| **IGT deck C** | 25.13 (7.18) | 26.72 (7.50) | 24.25 (6.92) | 22.92 (6.43) |
| **IGT deck D** | 29.35 (8.22) | 30.06 (8.38) | 29.44 (8.76) | 27.38 (6.80) |
| **IGT Block 1** | -3.47 (6.37) | -3.69 (5.83) | -4.22 (7.38) | -1.38 (5.37) |
| **IGT Block 2** | 1.06 (5.87) | 1.63 (5.77) | 1.77 (6.45) | -1.84 (4.12) |
| **IGT Block 3** | 3.64 (7.07) | 4.78 (8.07) | 3.18 (5.69) | 1.69 (6.92) |
| **IGT Block 4** | 4.02 (8.54) | 6.12 (9.24) | 2.96 (.60) | .92 (4.94) |
| **IGT Block 5** | 3.97 (7.72) | 4.90 (8.15) | 4.14 (8.13) | 1.23 (5.13) |
| **Net score** | 9.08 (21.76) | 13.78 (23.55) | 7.40 (22.04) | .61 (12.81) |
