# Supplementary material for: Eating Disorders Impact on Vigilance and Decision Making of a Community Sample of Treatment Naive Attention-Deficit/Hyperactivity Disorder Young Adults
Source: Front Psychiatry. 2018 Nov 6;9:531. doi: 10.3389/fpsyt.2018.00531 (PMC6232382; doi:10.3389/fpsyt.2018.00531)
Supplement: Supplementary file 3 [file Table_3.DOCX]

**Supplement.** Conner’s Continuous Performance Test .

Mean (standard deviation)

|  | ***Total*** | **Control Group** | **ADHD Group** | **ADHD+ED Group** |
| --- | --- | --- | --- | --- |
| **Omission** | 3.26 (13.12) | 1.63 (2.55) | 1.61 (2.07) | 10.69 (30.32) |
| **Comission** | 13.66 (8.36) | 11.96 (7.86) | 14.53 (8.57) | 16.23 (8.88) |
| **Standard Error** | 474.27 (175.11) | 461.48 (146.32) | 461.73 (162.64) | 531.84 (254.66) |
| **D Prime** | 70.14 (43.72) | 79.33 (43.43) | 66.60 (44.25) | 53.92 (40.66) |
| **Variability** | 605.30 (519.57) | 518.18 (163.54) | 604 (380.86) | 822.38 (1064.02) |
| **HRT** | 36346.88 (6411.55) | 36473.36 (6005.31) | 36188.23 (6846.77) | 36343.15 (7020.72) |
| **HRT Block Change** | .52 (1.05) | .38 (.86) | .6431 (.90) | .66 (1.66) |
| **HRT ISI Change** | 5.22 (2.74) | 4.99 (2.94) | 5.26 (2.52) | 5.69 (2.78) |
